# Fit Testing Retrofitted Full-Face Snorkel Masks as a Form of Novel Personal Protective Equipment During the COVID-19 Pandemic

**DOI:** 10.1017/dmp.2021.133

**Published:** 2021-04-19

**Authors:** Stephanie Toigo, Michel Jacques, Tarek Razek, Ewa Rajda, Sidney Omelon, Frederic Dankoff, Rami Tohme, Patricia Lefebvre, Dan L Deckelbaum

**Affiliations:** 1 Research Institute of the McGill University Health Center, Montréal, Canada; 2 Department of Occupational Health and Safety, McGill University Health Center, Montréal, Canada; 3 Department of Trauma Surgery, Montreal General Hospital, McGill University Health Center, Montréal, Canada; 4 Divisions of Infectious Diseases and Medical Microbiology, McGill University Health Centre, Montréal, Canada; 5 Department of Mining and Materials Engineering, McGill University, Montréal, Canada; 6 Department of Emergency Medicine, McGill University Health Center, Montréal, Canada; 7 Department of Infrastructure and Biomedical Engineering, McGill University Health Center, Montréal, Canada; 8 Department of General Support, Administration, and Performance, McGill University Health Center, Montréal, Canada

**Keywords:** personal protective equipment, fit test, COVID-19, snorkel mask, respirator

## Abstract

**Objective:**

Bottlenecks in the personal protective equipment (PPE) supply chain have contributed to shortages of PPE during the coronavirus disease 2019 (COVID-19) pandemic, resulting in fractures in the functionality of health-care systems. This study was conducted with the aim of determining the effectiveness of retrofitted commercial snorkel masks as an alternative respirator for health-care workers during infectious disease outbreaks.

**Methods:**

A retrospective analysis was performed, analyzing qualitative and quantitative fit test results of the retrofitted Aria Ocean Reef® full-face snorkeling mask on health-care workers at the McGill University Health Centre between April and June 2020. Historical fit test results, using medical-grade respirators, for health-care workers, were also analyzed.

**Results:**

During the study period, 71 participants volunteered for fit testing, 60.6% of which were nurses. The overall fit test passing rate using the snorkel mask was 83.1%. Of the participants who did not previously pass fit testing with medical-grade respirators, 80% achieved a passing fit test with the snorkel respirator.

**Conclusions:**

The results suggest that this novel respirator may be an effective and feasible alternative solution to address PPE shortages, while still providing health-care workers with ample protection. Additional robust testing will be required to ensure that respirator fit is maintained, after numerous rounds of disinfection.

With the emergence of the coronavirus disease 2019 (COVID-19) pandemic, challenges surrounding the availability and accessibility of medical-grade PPE were elucidated. Based on documented shortages of personal protective equipment (PPE) in China, it was important to anticipate such challenges in the rest of the world, including North America.^1^ However, due to bottlenecks in the supply chain for PPE and the global demand for these items, it became difficult to secure appropriate stocks.^2,3^ These shortages initially were observed in large hospitals in metropolitan centers; however, months later, these shortages are still prevalent in hospitals, nursing homes, and private medical clinics.^3^ The lack of PPE has contributed to relatively higher infection rates observed among health-care workers, accounting for up to 19.4% of the infected population in Canada. In 2019, there were approximately 1.3 million registered health-care providers in Canada, representing only 3.4% of the 2019 Canadian population, indicating a higher risk of infection observed among front-line health-care workers.^4,5^ This higher proportion of infection with severe acute respiratory syndrome coronavirus 2 (SARS-CoV-2) among health-care workers results in significant implications on the functionality of health-care systems as well as increased nosocomial infections.^1,6,7^

During a time where traditional medical-grade disposable PPE is of short supply, novel forms of reusable PPE, such as retrofitted commercial snorkel masks, may be an innovative and effective alternative. These masks have been designed as a contingency plan during infectious disease outbreaks such as the SARS-CoV-2 pandemic, where shortages of medical-grade PPE are an unfortunate reality. Having a reusable respirator may prevent health-care workers from having to reuse or extend the use of disposable PPE, as a means of conservation. Preliminary testing of these devices using qualitative and quantitative fit testing has proved to be as or more effective as using disposable N-95 respirators or equivalent.^8–11^ Although the snorkel mask is a full-face respirator, wearers did not experience any additional discomfort or obstruction of view, even when worn for prolonged periods of time, making it a feasible option to implement.^8–11^

While previous studies of snorkel mask respirators are important preliminary evaluations, we present the largest and most diverse series validating the fit of these respirator devices.^8–11^ We hypothesize that the retrofitted Aria Ocean Reef® full-face snorkeling mask will protect health-care workers from infectious SARS-CoV-2 droplets and aerosols and be a feasible solution to address potential PPE shortages.

## Methods

A retrospective analysis was performed, analyzing fit test results of health-care workers. Fit testing was initially conducted between April 17 and June 7, 2020 as a preparedness measure by the McGill University Health Center Hospitals (MUHC) as part of the PPE contingency plan during the COVID-19 pandemic. Fit test outcomes of the retrofitted respirator were analyzed along with historical fit test results of these employees using medical-grade respirators. Given the concern for potential shortages of PPE, health-care workers were proactive to secure an alternative respirator and volunteered to be fit tested with the snorkel mask. Participants included nurses, respiratory therapists, physicians, residents, patient attendants, technicians, and care advisors from various hospital departments. Based on previous fit test results of retrofitted snorkel masks, we estimated that the passing fit test rate would be approximately 96%.^8–11^ With this estimate and a 5% type I error and precision, a sample size of 59 would be required.

**Figure 1.**
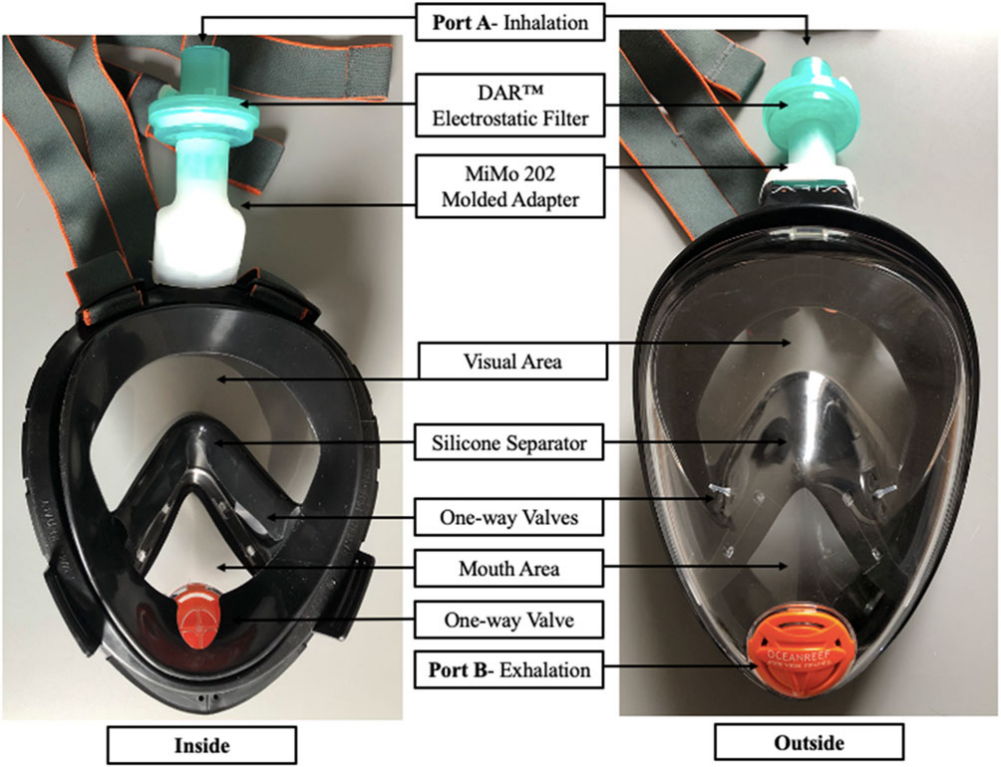
Retrofitted Aria Ocean Reef^®^ full-face snorkeling mask, for use as a personal protective equipment respirator outside of water.

The commercial mask undergoing fit testing is the Aria Ocean Reef® full-face snorkeling mask, which is available in 2 sizes (small and large) and obviates the need for separate ocular protection (Figure 1 **)**. For this mask to be used as a medical respirator, the snorkel is removed, and a moulded adapter (MiMo 202), created by AddiFab, Denmark, is connected between the mask and a filter with a ≥99.99% viral filtration efficiency, (DAR^™^ Electrostatic Filter) created by Medtronic, Canada.^12^ Using techniques outlined by Nelson Laboratories Inc®, a liquid suspension of the bacteriophage phiX174 is aerosolized at a constant flow rate toward the filter to test viral filtration efficiency. This test allows for comparison of the viral control counts and the filter effluent counts to determine the viral filtration efficiency.^13^ Inhalation occurs through Port A, and exhalation occurs through one-way valves at Port B. Between the mouth area and visual area, there is a silicon separator minimizing dead space in the mask with a unidirectional flow of air through the mask. This separator prevents fogging within the mask, which ensures an unobstructed visual field for the user, adding to the comfort of the mask.

Both qualitative and quantitative fit tests were conducted on the Aria Ocean Reef® snorkeling mask without disinfection as well as four masks that endured various types of disinfection procedures. The qualitative fit tests were conducted using a Bitrex® fit-testing kit. This requires the person undergoing testing to don a respirator that provides the best fit and comfort for them. The test administrator will then place the test hood over their head and aerosolize the Bitrex® test solution into the hood. If the user is able to taste the bitter substance while performing a variety of exercises, including normal breathing, deep breathing, grimacing, talking (counting to 30), turning their head side-to-side (10 times), flexing and extending the neck (10 times), and bending over at the hip (10 times), then that indicates that the mask did not achieve an adequate seal and that they failed the fit test. Once completing all maneuvers without detecting the bitter taste, the mask is lifted slightly while still under the hood to confirm the presence of the bitter taste.^14–16^ Quantitative fit testing was conducted using a PortaCount® respirator fit-testing device, which measures total aerosol penetration.^8^ The same seven fit test exercises used for the Bitrex® testing method were used for the PortaCount®. For each of these exercises, a fit factor was established, which is the ratio of the ambient particle concentration to the in-mask particle concentration.^14–16^ A passing fit test is achieved if the participant obtains a fit factor of 100 or more, anything below this value is insufficient to achieve a proper seal and block out microbial particles.^15,16^

## Results

During the study period, 71 participants volunteered for fit testing. The majority of participants were nurses 43 (60.6%) and respiratory therapists 16 (22.5%). These participants primarily worked in the intensive care unit 45 (63.4 %). Most participants completed a qualitative fit test using the Bitrex® testing kit 67 (94.4%). Of those who completed this testing, the passing rate for the snorkel mask was 82.1%. A smaller proportion of participants were tested using a quantitative method by means of a PortaCount® device (5.6%). All of the participants who were tested using this method had a passing score. Over half of the participants found a better fit using the smaller size of the Aria Ocean Reef® mask 38 (53.5%) and with this size of mask, the proportion that passed either the qualitative or quantitative testing was approximately 95%. Fewer participants found that the larger size was a more comfortable fit 18 (25.4%), and similarly, the proportion that passed the fit testing with this size of mask was approximately 95%. There were 14 participants who did not have their preferred mask size recorded, and the proportion that passed fit testing among this group was much lower (42.9%). One participant tried both mask sizes and did not pass the fit-testing with either size. Overall, over 83% of those who underwent fit testing with the snorkel mask achieved a passing score (Table 1).

**Table 1.** Fit test results using the Aria Ocean Reef® retrofitted full-face respirator and historical fit test results using medical-grade respirators

	Fit test results of the Aria Ocean Reef®		
	Participants	Pass	Fail
	————————————————— *N* (%) ————————————————		
Fit test results: Aria Ocean Reef®	71 (100)	59 (83.1)	12 (16.9)
Fit test procedure: Aria Ocean Reef® *(n = 71)*			
Bitrex®	67 (94.4)	55 (82.1)	12 (17.9)
PortaCount®	4 (5.6)	4 (100)	0 (0)
Model of Aria Ocean Reef® tested *(n = 71)^a^*			
Small	38 (53.5)	36 (94.7)	2 (5.3)
Large	18 (25.4)	17 (94.4)	1(5.6)
Both	1 (1.4)	0 (0)	1 (100)
Not indicated	14 (19.7)	6 (42.9)	8 (57.1)
Fit test results: Medical-grade respirators (*n = 65*)^bc^	65 (90.3)	53 (81.5)	12 (18.5)
Medical-grade: all failed	5 (7.7)	4 (80.0)	1 (20.0)
Medical-grade: passed with one respirator	25 (38.5)	19 (76.0)	6 (24.0)
Medical-grade: passed with >1 respirator	35 (53.8)	30 (85.7)	5 (14.3)
Mean ± SD (min-max) Median [IQR]		2.2 ± 0.43 (2.0-3.0) 2.0 [2.0, 2.0]	2.6 ± 0.55 (2.0-3.0) 3.0 [2.0, 3.0]

a Before testing, participants were able to try both sizes of the retrofitted respirator to select the one that provided the most comfort and then underwent testing with that size of respirator.

b A subsample of 65 participants had previously undergone fit testing using medical-grade respirators. The fit test results of these respirators have been presented with those same participants’ fit test results using the Aria Ocean Reef® respirator.

c Types of disposable N-95 respirators and reusable half face piece respirators previously tested: 3M^™^ 1860/ 1860S/ 1870+/ 8200/ 82100, Moldex® 1510/ 1511/ 1512/ 1517, 3M 7501/ 7502/ 7503 and Champak^©^ F550.

Abbreviation: IQR, interquartile range.

Retrospective fit test results of these participants, using medical-grade respirators were obtained to analyze alongside the snorkel mask fit test results. Through routine Occupational Health and Safety practice, 65 of our participants had previous fit test results with medical-grade respirators, using either the Bitrex® testing kit or the PortaCount® device (Table 1). More than half of the participants 35 (53.8%) previously passed fit testing with more than one of the medical-grade respirators. There were five participants that failed all fit testing with traditional medical-grade respirators, of which, 80% of them achieved a successful fit test with the snorkel respirator. However, there is no statistically significant relationship between an unsuccessful fit test with a medical-grade respirator and achieving a passing fit test with the snorkel respirator.

Two participants, that passed all initial fit tests with the snorkel respirator, underwent additional fit testing using four respirators that had endured various disinfection practices. The disinfection practices included 2-hour submersion in dilute bleach solution, 5-hour submersion in dilute bleach solution, and 10 cycles (3 hour each) of vaporized hydrogen peroxide. These participants passed fit testing with all of the disinfected respirators.

## Discussion

The ongoing COVID-19 pandemic has created a strain on the PPE supply chain due to the high demand for medical-grade PPE. Evaluating alternative forms of PPE is crucial to serve as a contingency plan. Having reusable respirators, such as the retrofitted full-face snorkel mask, not only will be useful during the current pandemic but will allow health-care facilities to be better equipped for future public health emergencies that will inevitably happen.^3^ However, rigorous testing of these devices needs to be conducted to ensure the respirator is effective at protecting health-care workers from biohazardous material, especially after undergoing multiple rounds of cleaning and disinfection.^3,16^

Four previous studies have used qualitative and quantitative fit testing to examine the ability of full-face snorkel masks to protect health-care workers, without compromising comfort.^8–11^ These studies have demonstrated positive outcomes from fit testing, however, were limited by very small sample sizes (≤10 participants), making their results less generalizable to a more diverse population of health-care workers that have varying facial morphologies.^8–11^ Our study is the largest to date that has fit tested retrofitted snorkel masks, based on sample size calculations, our sample of 71 participants is sufficient to reflect an accurate representation of the target population, and to observe the snorkel mask’s ability to perform as a protective barrier between the hospital environment and the user.

The viral filtration efficiency of the filter used in the retrofitted snorkel mask is approximately 99.99% and in combination with a passing fit test, the snorkel mask is a highly effective PPE contingency option to minimize health-care worker’s encounters with harmful microbes, when standard N-95 respirators are in short supply. The positive fit test results that were observed among our study as well as those previously published, suggest that this novel respirator may be an effective and feasible solution for the lack of available PPE, while still providing health-care workers with ample protection.^8–11^ The participants in our study, as well as those in previous studies, indicated that the retrofitted respirator did not cause any discomfort or hinder their ability to perform their duties. To further support the use of this alternative PPE, national regulatory board approvals are being sought.

Limitations of this study include the type of fit testing performed and those inherent to any retrospective analysis. The majority of our fit tests were performed using a qualitative method, which is more subjective compared with quantitative tests, as it relies on the participant’s ability to detect a bitter taste to indicate a failed test. Whereas quantitative testing provides a fit factor that is a numerical value of how well the mask fits against the user’s face, which is calculated by finding the ratio of aerosols outside the mask to that inside.^15,16^ Additionally, some fit testing guidelines have recommended using quantitative fit testing for full-face respirators instead of qualitative. This is based on the assigned protective factor (APF) that the Occupational Health and Safety Administration (OHSA) determines for each respirator, which is the measure of minimization of ambient contaminant concentration while wearing the respirator. Full-face respirators typically have an APF of 50, indicating that the ambient contaminant concentration inside the respirator will be reduced by at least 50 times, while worn.^15,16^ There is no qualitative testing currently available that can determine a passing score for a respirator with an APF greater than 10.^15,16^

While our study primarily used qualitative fit testing, which remains a standardized fit testing method, we did perform a small number of quantitative fit tests, all of whom passed. Although previous research has indicated that quantitative fit testing has a higher test specificity and produces less false-positive results compared with qualitative testing, there is still a substantial amount of agreement between the two testing methods (κ = 0.63).^14^

## Conclusions

Overall, our preliminary study was able to highlight the effectiveness of the Aria Ocean Reef® retrofitted snorkel mask for use as a full-face respirator, by achieving a high proportion of passing fit test results as well as user-reported comfort. It also suggested that this device might be an effective alternative solution of PPE for those who were unable to achieve a passing fit test with medical-grade respirator masks, during times of PPE shortages. Further research will be needed to obtain a quantifiable fit-factor for the fit testing of this device. Although passing fit tests were observed using respirators that underwent disinfection, more rigorous testing will need to be conducted after numerous rounds of disinfection to ensure that a tight seal around the user’s face is maintained. Additionally, biomedical and material engineering studies are necessary to ensure component integrity after multiple uses and disinfection cycles. The retrofitted full-face snorkel mask proves to be an effective, novel form of PPE for contingency use during the ongoing COVID-19 pandemic and may provide better preparedness for future pandemics that prompt shortages of single-use PPE.
